# The oral sensory organs in *Bathochordaeus stygius* (Tunicata Appendicularia) are unique in structure and homologous to the coronal organ

**DOI:** 10.1186/s12983-023-00518-8

**Published:** 2023-12-15

**Authors:** Mai-Lee Van Le, Lisa-Marie Müller, Thomas Stach

**Affiliations:** Humboldt-Universität zu Berlin Vergleichende Elektronenmikroskopie, Philippstraße 13, 10115 Berlin, Germany

**Keywords:** Larvacea, Lateral line system, Urochordates, Sense organ, Secondary receptor cells

## Abstract

**Background:**

Appendicularia consists of approximately 70 purely marine species that belong to Tunicata the probable sister taxon to Craniota. Therefore, Appendicularia plays a pivotal role for our understanding of chordate evolution. In addition, appendicularians are an important part of the epipelagic marine plankton. Nevertheless, little is known about appendicularian species, especially from deeper water.

**Results:**

Using µCT, scanning electron microscopy, and digital 3D-reconstruction techniques we describe three pairs of complex oral sensory organs in the mesopelagic appendicularian *Bathochordaeus stygius*. The oral sensory organs are situated at the anterior and lateral margin of the mouth and inside the mouth cavity. A single organ consists of 22–90 secondary receptor cells that project apical cilia through a narrow hole in the epidermis. The receptor cells are innervated by branches of the second brain nerve.

**Conclusions:**

Based on position, morphology, and innervation we suggest that the oral sensory organs are homologues of the coronal organs in other tunicates. We discuss the hypothesized homology of coronal organs and the lateral line system of primary aquatic vertebrates. The complex oral sensory organs of *B. stygius* are unique in tunicates and could be adaptations to the more muffled environment of the mesopelagic.

**Supplementary Information:**

The online version contains supplementary material available at 10.1186/s12983-023-00518-8.

## Background

Although in some regions the purely marine appendicularians are the second most abundant zooplankton group after copepods [19, 21, 26], relatively little is known about most of the 70 recognized species. Scientific studies [e.g. 13, 27, 38, 40, 46, 52] have focused on the model species *Oikopleura dioica*, while progress on other species notably from deeper waters lagged behind.

Appendicularians possess several sensory systems that enable them to coordinate their complex behaviors in relation to their planktonic environment. One of these sensory systems is the so-called coronal organ that is known from light- and electron microscopic investigations in *O. dioica* and *O. albicans* [40, 47, 48]. There, the coronal organ consists of single secondary sensory cells arranged in a ring surrounding the mouth. Lohmann [28] reports similar arrangements for species in oikopleurids, fritilariids, and kowaevskiids i.e. the coronal organ is present in all three appendicularian families. For *Fritillaria haplostoma*, Lohmann describes two additional large cells behind the circumoral ring inside of the anterior mouth cavity. In the species description of the mesopelagic *Bathochordaeus stygius* Garstang [18] mentions a ring of outer sensory cells and a pair of larger sensory cells inside of the mouth cavity. Because he was unable to use more advanced microscopic techniques Garstang’s statements were rather tentative, and he cautiously admits that no “bristles” were visible in the outer sensory cells but “striations” indicated that a “tuft of bristles'' was present in the larger inner sensory cells. Garstang suggested that this arrangement was similar to specimens in the genera *Kowalevskia* and *Fritillaria*. Nevertheless, in discussing other morphological features such as the oikoplastic fields or the lack of oral gland cells and subchordal cells Garstang cautiously suggests affinities of *B. stygius* to oikopleurids.

The coronal organ in tunicates is considered to be homologous to the lateral line system of primary aquatic vertebrates [e.g. 11, 32–34]. While the sensory cells in the lateral line are hair cells, with a single apical cilium in most cases surrounded by microvilli [4, 36, 54], the sensory cells of the coronal organ show a variety of morphologies: they can be cells with a single apical cilium, two cilia, or several cilia; microvilli can be present or absent [48]. Conversely, the coronal organ is always situated around the mouth openings in ascidians, appendicularians, and thaliaceans except for salps where the coronal organ seems to be entirely lacking [48]. In vertebrates, however, the sensory cells of the lateral line system are arranged along various lines on the skull and the trunk. They can be present as single cells and found in the inner ear as well [e.g. 3, 14, 41]. Thus, the documented disparity is high at the cellular level in tunicates and at the organ level in craniates. Here, we show that the coronal organ of the mesopelagic giant appendicularian *Bathochordaeus stygius* does not consist of a circumoral ring of secondary sensory cells but instead of three pairs of more complex oral sensory organs. This unique arrangement and anatomy may reflect the sensory requirements in a habitat that is unusual for appendicularians and demonstrates evolutionary diversity on several organismal levels.

## Results

**Fig. 1:**
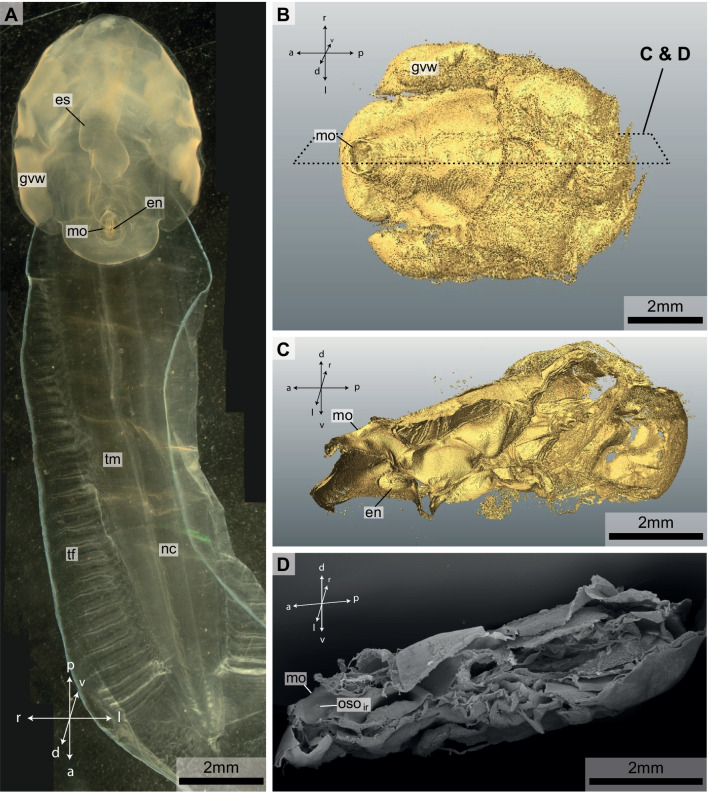
**A** Light micrograph of a specimen of *Bathochordaeus stygius*. **B** µCT-isosurface image of the specimen shown in A in dorsal view. Dashed rectangle indicates cutting plane from figures (**C** and **D**). **C** µCT-isosurface image of the trunk of the animal in lateral view from the left side. The left half of the animal was digitally removed along the mid-sagittal plane to show inner structures. **D** Scanning electron micrograph of the half of the same specimen sectioned along the mid-sagittal plane. Note the mouth opening (mo) to the left and the inner oral sensory organ of the right side (oso_ir_) inside the mouth cavity. In **B**, **C** and **D** orientation is anterior to the left posterior to the right. es—esophagus, en—endostyle, mo—mouth opening, nc—nerve chord, tm—tail muscle, oso—oral sensory organ, gvw—genito-visceral wing, tf—tail fin

**Fig. 2:**
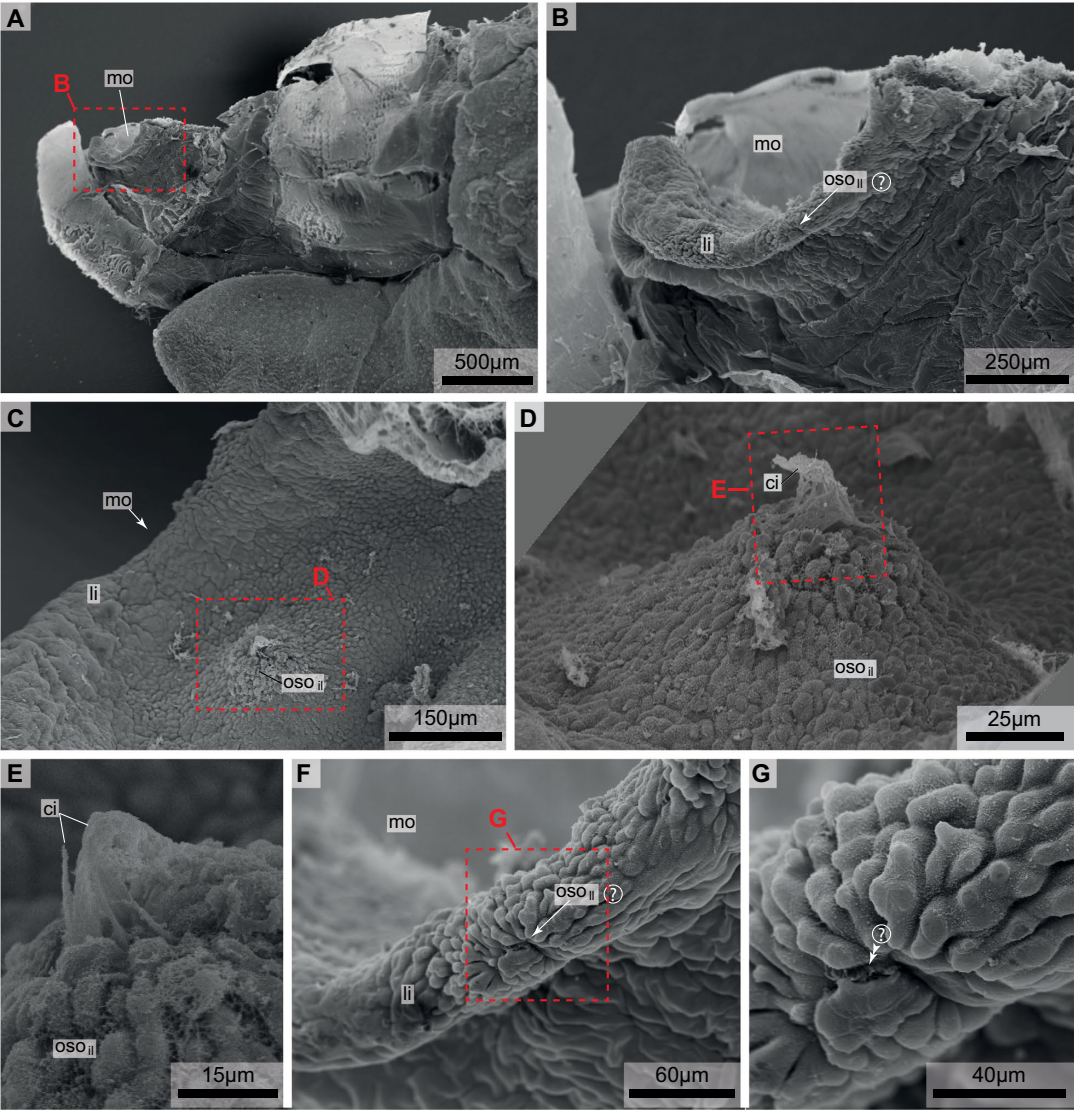
Scanning electron micrographs of *Bathochordaeus stygius.* Specimen cut along the sagittal mid-plane. **A** Oblique dorsal view anterior to the left. **B** Oblique dorsal view of mouth opening. **C** Lateral view of the anterior mouth cavity. Anterior to the upper right of the image. **D** Interior oral sensory organ of the left side. **E** Ciliary tuft of the interior oral sensory organ of the left side. **F** Lateral lip. Note irregular shape of epidermis cells. **G** Magnification of F depicting possible cilia of the lateral oral sensory organ of the left side (double arrowheads). ci—cilia, li—lip, mo—mouth, oso_il_—inner oral sensory organ of the left side, oso_ll_—possible lateral oral sensory organ of the left side

**Fig. 3:**
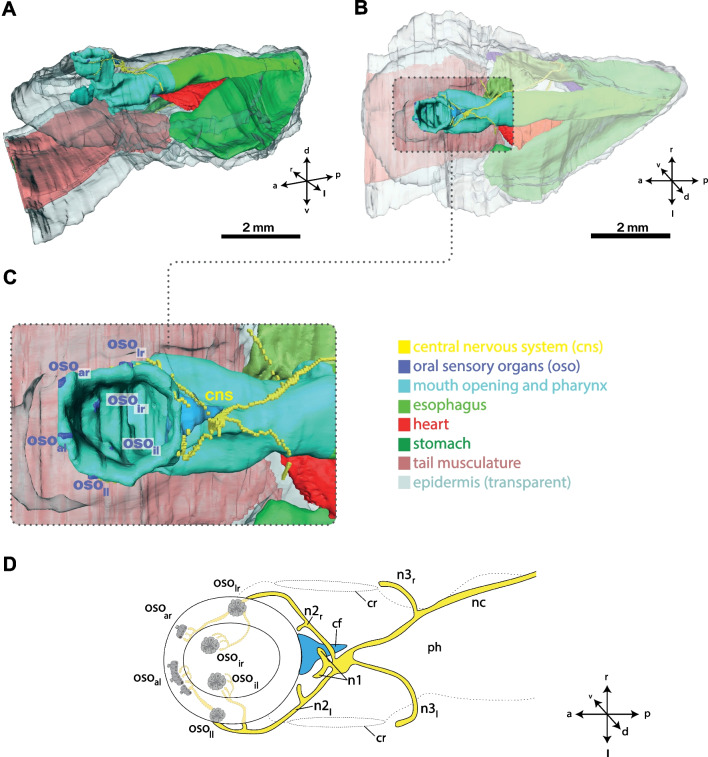
Digital 3D-Reconstruction of the anatomy of *Bathochordaeus stygius*. **A** Complete trunk in oblique view from the left. Epidermis is semi-transparent to show inner organs. Note the dorsally oriented mouth opening. **B** Dorsal view with dotted rectangle highlighting mouth pharynx and central nervous system. **C** Enlarged dorsal view of the rostral region highlighted in (**B**). Note the symmetrical arrangement of sensory organs on the margin of the mouth and the inner rostral pharynx. **D** Schematic illustration of the oral region and nervous system in *B. stygius* with distribution of the oral sensory organs and innervating nerves. cf—ciliary funnel, cr—ciliary ring of gill slit, n2_l/r_—second brain nerve of left/right side, n3_l/r_—third brain nerve of left/right side, nc—nerve chord, oso_al/r_—anterior oral sensory organ of the left/right side, oso_ll/r_—lateral oral sensory organ of the left/right side, oso_il/r_—inner oral sensory organ of the left/r side, ph—pharynx

The trunk length of the two specimens of *Bathochordaeus stygius* examined for the present study measured 7 mm and 9 mm respectively (Fig. 1). The mouth opening is dorsally directed and almost circular with a diameter of approximately 0.6 mm (Figs. 1A, B, 2A, B). The anatomical reconstructions based on the µCT-data (Fig. 1B, C) and based on the serial histological sections (Fig. 3A–C) agreed with the findings and images of the original species description [18]. No indication of individual malformation could be detected.

In scanning electron microscopic aspects, epidermis cells in the mouth region are of somewhat irregular ovoid shape measuring approximately 15 µm × 10 µm (Fig. 2). In general, these epidermis cells feature no apical cilia. On the margin of the mouth, we could not detect cilia using scanning electron microscopy. Occasionally, debris remained attached to the epidermis. Laterally, inside the mouth cavity, epidermis cells bulge inwards forming a small mound of approximately 100 µm diameter (Fig. 2C–E). In the center of this mound a tuft of cilia projects about 15 µm above the apical surface of the epidermis cells.

**Fig. 4:**
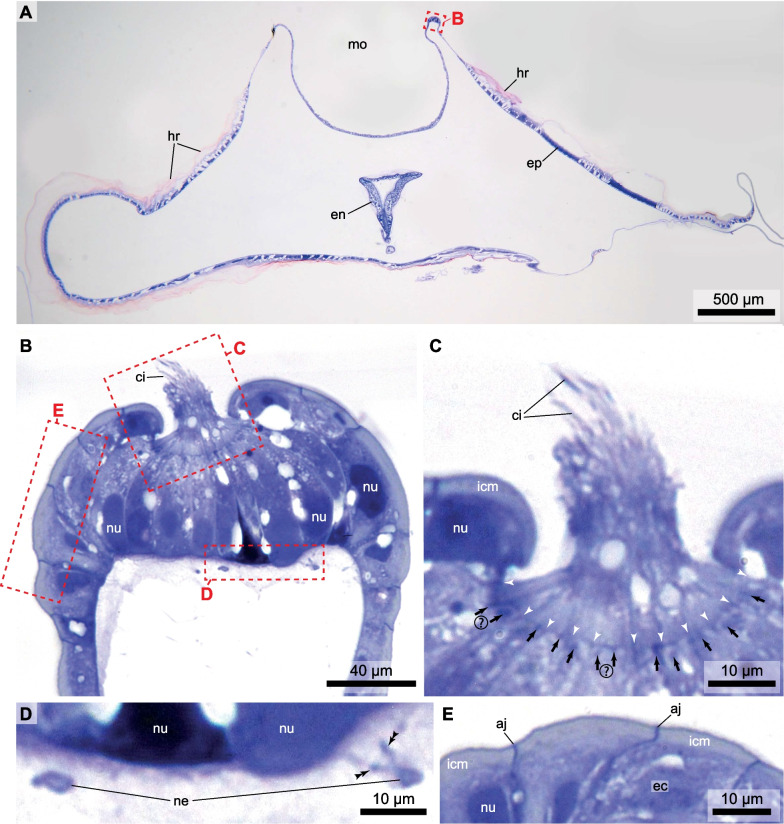
**A** Light micrograph of a transverse section of *Bathochordaeus stygius*’ oral region displaying the dorsal mouth opening with the lateral sensory organ on the right side of the mouth (mo). **B** Light micrograph of the lateral right oral sensory organ with frames highlighting the cilia, the epidermal cells, and the neurites enlarged in (**C**, **D**, and **E**). **C** Bundle of cilia (ci) emerging between epidermis cells. White arrowheads point to cell contacts. Black arrows point at apical dark areas probably (questionmarks: possibly) rootlets of cilia. **D** Neurites (ne) contacting the sensory cells (double arrowheads). **E** Apical part of epidermis cells with intracellular matrix (icm). aj—apical junctions, ec—epidermis cell, en—endostyle, ep—epidermis, hr—house rudiment, icm—intracellular matrix, ne—neurites, nu—nucleus, sc—sensory cell

**Fig. 5:**
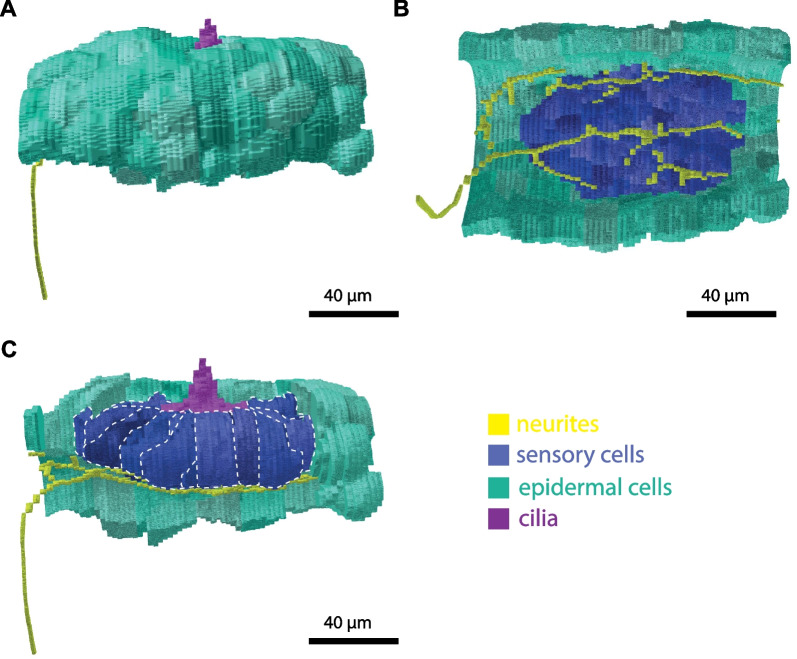
Reconstruction of the lateral oral sensory organ of the right side. **A** Lateral view from the left showing neurite (yellow) approaching the oral sensory organ and the apical cilia (purple) emerging between the epidermis cells (turquoise). **B** Ventral view showing the innervation of sensory cells (blue) via a network of neurites. **C** same perspective as in A with epidermis cells removed from the left side showing the extension of the sensory cells. Dashed lines mark boundaries of sensory cells

From serial sections, light micrographs were recorded and 3D reconstructions of the entire trunk, the nervous system, target cells (Fig. 3), and the six oral sensory organs (Figs. 3C, D, 4, 5) of one individual of the giant appendicularian *Bathochordaeus stygius* were produced. One of the oral sensory organs, the lateral oral sensory organ of the right side, was reconstructed in detail. For the remaining five oral sensory organs, we analyzed their histology. In addition, we reconstructed the shapes, numbers, and relative positions of the nuclei of the sensory cells in 3D for these five oral sensory organs (Additional file 1: Figures S1, S2).

**Fig. 6:**
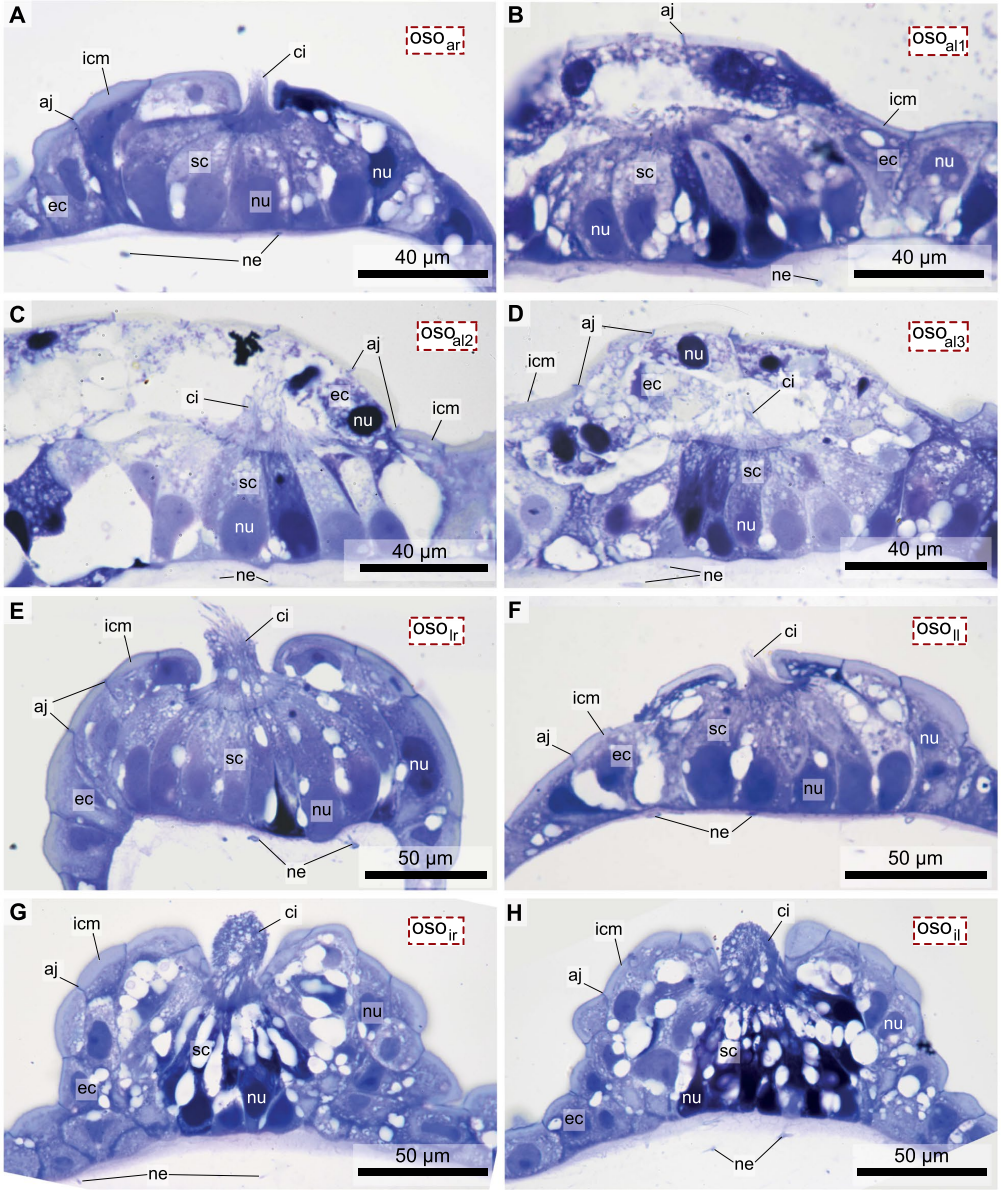
Light micrographs of cross sections of all oral sensory organs (oso) in a specimen of *Bathochordaeus stygius.* oso_ar/l_: anterior oral sensory organ of the right/left side, oso_lr/l_: lateral oral sensory organ of the right/left side, oso_ir/l_: inner oral sensory organ of the right/left side. Note that oso_al_ contains three clusters of sensory cells, oso_al1-3_. The apical openings in the epidermis covering of oso_al1-3_ are depicted in Additional file 2: Figure S2. aj—apical junctions, ec—epidermis cell, ep—epidermis, hr—house rudiment, icm—intracellular matrix, ne—neurites, nu—nucleus, sc—sensory cell

The reconstruction of the entire trunk visualizes the anatomy of inner organ systems including the nervous system consisting of a brain and the peripheral nerves (Fig. 3). The brain is situated posterior to the dorsally directed mouth opening. Branches of the second brain nerve project anteriorly around the mouth and contact three oral sensory organs on each side (Figs. 3C, D, 4, 5). Two of these oral sensory organs are located on the rim of the mouth opening, on each side of the mouth. One of them is situated at the anterior edge (anterior oral sensory organ) the other about 250 µm posterior to the former (lateral oral sensory organ). An additional third oral sensory organ is situated on the inner wall of the anterior mouth cavity (inner oral sensory organ; Figs. 2, 3C, D, 6).

We reconstructed the three-dimensional anatomy of the lateral oral sensory organ on the right side in cellular detail based on every 2 µm-section of the complete series, 59 sections together (Figs. 4, 5). The right lateral oral sensory organ is of elongated ellipsoid shape being approximately 80 µm long, 60 µm wide, and 50 µm high. It rests on the epidermal basement membrane and is covered by epidermis cells which leave only a small opening in the middle of the organ. Cilia of the sensory cells pass through this circular opening that has a diameter of approximately 10 µm (Figs. 4B, C, 5A, C). At the basal side the neurite of the second brain nerve approaches the lateral oral sensory organ and branches repeatedly (Figs. 4B, D, 5B, C).

The entire lateral oral sensory organ of the right side contains 67 sensory cells. These cells are of an elongated flask shape being about 50 µm tall (Fig. 4B). The sensory cells are polar in organization. Their bases rest on the epidermal basement membrane and apically, a darker band marks the apical adherens zone (Fig. 4C). Moreover, the cells are equipped apically with sensory cilia (Figs. 4B, C, 5A, C). They appear monociliated, although light microscopical resolution is unsuitable to prove this beyond doubt (Fig. 4C). With 15 µm of breadth at the base in cross sections the cells are much wider than at the apical side where the apex of the cells passes through a circular opening of the epidermis (Fig. 4A, C). Here, the cells measure barely 2–3 µm. The nuclei of the sensory cells are situated in the basal part of the cells. They are oval in cross section with a height of 7 µm and a breadth of 3 µm (Fig. 4B). The cytoplasm stains moderately to darkly but evenly with toluidine blue. In addition, there are empty appearing vesicles of different sizes present in the cytoplasm of the entire cells (Figs. 4B, C, 6).

The neurite branches of the second brain nerve penetrate the basement membrane and approach the basal sides of the sensory cells closely (Fig. 4D). While the adjacent epidermis cells overarch the sensory cells (Fig. 4B, E), they are clearly distinguishable from the sensory cells by their conspicuous apical intracellular matrix (Fig. 4B, C, E).

Generally, the epidermis cells are roughly cuboid in cross section, containing a prominent central nucleus and a lightly staining cytoplasm.

The detailed reconstruction of the exemplary lateral right oral sensory organ was compared to sections of the remaining five oral sensory organs from the same series of sections (Fig. 6). This comparison demonstrated that histologically, all oral sensory organs are built similarly. They all consist of a cluster of elongated, flask-shaped secondary sensory cells, that are innervated by branches of the second brain nerve. All secondary sensory cells are polar in organization and possess apical cilia that project through an opening in the overlying epidermis cells. Nevertheless, the reconstruction of the nuclei of sensory cells in all remaining oral sensory organs showed that the numbers of sensory cells differ significantly between the oral sensory organs, varying between 22 cells in the left lateral oral sensory organ and 90 in the anterior left oral sensory organ (Table 1, Additional file 1: Figure S1). Moreover, the anterior left oral sensory organ (oso_al_) consisted of three clusters of sensory cells. These clusters were not separated by epidermis cells, yet each possessed its own ciliary tuft and epidermal opening (Figs. 3D, 6B, C, D, Additional file 1: Figure S1, S2).

**Table 1:**
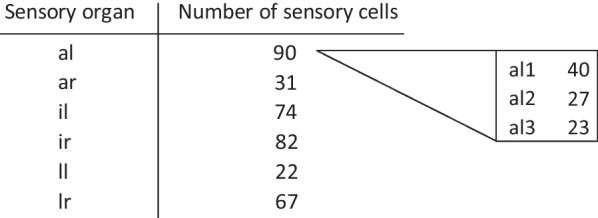
Number of sensory cells derived from 3D reconstructions of nuclei

Note that oso_al_ contains three clusters of sensory cells for which numbers are listed additionally

al/r, anterior left/right; il/r, inner left/right; ll/r, lateral left/right

## Discussion

### Receptor cells in the oral sensory organs of *B. stygius* are secondary sensory cells

Three oral sensory organs are located at the anterior and lateral rim of the mouth and in the mouth cavity in *Bathochordaeus stygius* on each side. These oral sensory organs are composed of 22–90 sensory cells that project an apical cilium through a narrow opening left by the non-ciliated epidermis cells that otherwise cover the oral sensory organs. The oral sensory organs are innervated by branches of the ventral ramus of the second brain nerves on either side. The second brain nerves consist of a single ramified neurite emerging from the perikarya of a neuron located laterally in the anterior forebrain on each side [55]. Thus, even without confirmation by transmission electron microscopy it is reasonably clear that the receptor cells in the oral sensory organs are secondary sensory cells.

### Oral sensory organs in *B. stygius* are homologues of coronal organs in other appendicularians

In the original species description, Garstang [18] describes sensory cells on the lower lip of *B. stygius*. He observed a striation but admitted that the resolution of his microscopic images did not allow to ascertain whether apical “bristles” were indeed present. Different from our findings, Garstang describes these sensory structures as arranged continuously around the lower lip and extending with single cells to the upper lip. In our high-resolution micrographs of serial sections, as well as in our resulting 3D reconstruction, and the SEM analysis, we could not detect such an arrangement. In accordance with our findings, Garstang reported a pair of “inner sensory cells” inside of the mouth cavity larger than the ones on the lower lip [18]. For these, he explicitly ascertained the presence of “apical striations” that he interpreted as “bristles”. His observations however were probably made on specimens fixed in Bouin’s fixative, cleared in glycerol, and with a microscope “without cover slip”. Unfortunately, no further details are given and we are therefore unable to gauge the limits of resolution in his study. In his drawings (Figs. 9, 11 in [18]), the “inner sensory cells” correspond in position, size, and shape to the inner oral sensory organs found in our specimens.

In the model organism *Oikopleura dioica*, the best known appendicularian species, several presumably sensory systems are known from the oral region [see 41]. Of these, only the ventral sensory organ comprises a compact organ, consisting of numerous receptor cells similar to the oral sensory organs of *B. stygius* [7]. However, the ventral sensory organ of *O. dioica* is located ventrally in the anterior lateral epidermis. Moreover, the receptor cells of the ventral sensory organ are primary sensory cells, the neurites of which constitute a major part of brain nerve one. Sensory cells in the upper lip in *O. dioica*, are two enigmatic receptor cells situated in the dorsal rim of the mouth opening and are innervated by the dorsal branch of brain nerve 2. The upper lip cells do not possess cilia, and because of their position and flattened morphology, Olsson et al. [42] suggested a mechanosensory role.

In *O. dioica* the ventral branch of brain nerve 2 innervates sensory cells in the lower lip and anterior pharynx [42], that, according to a more recent study, form a circumoral ring and collectively are termed coronal organ [48]. These cells are individual secondary receptor cells with several apical cilia of different lengths [34, 40, 48]. The oral sensory organs of *B. stygius* are similar to the ventral sensory organs of *O. dioica* in that they form compact organs composed of numerous receptor cells [7]. These receptor cells on the other hand are primary receptor cells innervated by the first brain nerve in *O. dioica*, whereas they are secondary receptor cells in the case of the oral sensory organs of *B. stygius*. Moreover, receptor cells of the oral sensory organs are innervated by the ventral branch of brain nerve 2. These latter characteristics are similar to the receptor cells of the coronal organ in *O. dioica*. Thus, the correspondences in innervation pattern, cell type, and location in the lower lip and anterior pharynx clearly support the hypothesis that the oral sensory organs of *B. stygius* are homologues of the coronal organ in *O. dioica* (see Table 2).

**Table 2:** Comparison of characteristics of the oral sensory organs (oso) of *Bathochordaeus stygius* with sensory structures around the mouth opening in *Oikopleura dioica* (highlighted in bold). See discussion for details

	Type of organ	Type of sensory cells	Apical cilia on sensory cells	Innervation
OSO	Compact organ	Secondary sensory cells	Present	2nd brain nerve, ramus ventralis
**Coronal organ**	**Row of individual cells**	**Secondary sensory cells**	**Present**	**2nd brain nerve, ramus ventralis**
**Ventral organ**	**Compact organ**	**Secondary sensory cells**	**Present**	**1st brain nerve**
**Upper lip cells**	**Two individual cells**	**Secondary sensory cells**	**Absent**	**2nd brain nerve, ramus dorsalis**

Martini [35] found sensory cells surrounding the mouth opening in *Fritillaria pellucida*. He described each cell as bearing a row of long cilia which “fused together” formed a “sensory sheet”. Bone et al. [8] demonstrated that (at least) the receptor cells in the lower lip were secondary sensory cells. Though these cells differ from the cells in our specimen in being multiciliated, they are similarly contacted by nerve fibers at their bases.

### Oral sensory organs in *B. stygius* are homologues of coronal organs in other tunicates

Several sensory organs possessing receptor cells with apical cilia are present in ascidians such as the cupular organ [9] the coronal organ [12, 33] or the capsular organ [31]. Only the coronal organ features secondary sensory cells [e.g. 34]. Moreover, while the cupular organ and the capsular organ are situated in the atrial cavity, the coronal organ is located on the oral tentacles corresponding in position to the mouth opening in appendicularians. Precise correspondences between brain nerves in ascidians and appendicularians are unclear [10], nevertheless sensory cells in the coronal organs of ascidians [e.g. 29] and in the oral sensory organ of *B. stygius* [present study 55], and the lower lip and pharyngeal cells in *O. dioica* [42, 48], are innervated by paired anterior brain nerves. Cupular and capsular organs on the other hand are innervated by paired posterior brain nerves [29, 30]. Thus, we suggest that these similarities support the hypothesis that the oral sensory organs in *B. stygius* are a homologue of the coronal organ of ascidians.

Differences remain as the sensory cells in the coronal organs in most ascidian species are multiciliated cells [48]. But there are exceptions, like some stolidobranchiate ascidians or the thaliacean tunicates *Pyrosoma atlanticum* and *Doliolum nationalis* [48]. Interestingly, the multiciliated secondary sensory cells in the coronal organ of *Ciona intestinalis* develop from a monociliated condition during ontogeny [48].

### Further considerations of potential homologues within chordates

Manni et al. [32] and Rigon et al. [47, 48] suggested that the tunicate coronal organ corresponds to the lateral line system of primarily aquatic craniates. Receptor cells in the lateral line system are secondary receptor cells, most with a single apical cilium surrounded by microvilli (so called hair-cells or neuromasts) [e.g. 15, 17, 51]. In fishes, neuromasts are dispersed throughout the epidermis mainly along the sides and on the head where they also run along the lower lip. Secondary sensory cells with an apical cilium surrounded by microvilli exist in the epidermis of cephalochordates as well [24, 25, 50]. Some are found dispersed individually in the epidermis but some are also found as so-called oral spines surrounding the mouth in larval stages. The latter position is similar to the oral tentacles and lips in other chordates and the similarities support the hypothesis of homology of these structures. Moreover, genes expressed in placodes—developmental precursors of the lateral line system—such as *eya* or *six1* [6, 23, 43] are also expressed in the oral region of developmental stages of *Ciona intestinalis* [44] and *Oikopleura dioica* [5]

### Functional consideration

Receptors in the lateral line system of vertebrates are mechanosensory or electrosensory [e.g. 15, 54]. The position around the mouth suggests that the oral sensory organs in *Bathochordaeus stygius* play a role in sensing food particles. For the receptor cells in the coronal organ of *Oikopleura dioica*, Rigon et al. [48] claimed a mechanosensory function. However, no neurophysiological experiments have been conducted and the mechanosensory faculty is based on structural similarities to ascidian coronal organs and vertebrate neuromasts and the suggested homology between the coronal organ and lateral line system. At the entrance of the digestive system a chemosensory capacity would also be plausible and numerous cases are described where chemoreceptors are structurally similar to the oral sensory organs in *B. stygius* [e.g. 1, 22]. In addition, more recent studies in marine invertebrates emphasize that sensory cells can be—and during evolution probably were—multimodal [2, 39].

Most observations of appendicularians are reported from the euphotic zone [28] although there might be a bias due to the technical difficulty of sampling deeper water [e.g. 19]. There are several species described that seem to occur exclusively in deeper layers [e.g. 19]. *B. stygius* is a mesopelagic form [18, 20]. It regularly occurs at depths below 200 m and descends at least as far as 1000 m. At these depths, forces exerted via currents and waves are less than in upper layers [e.g. 37, 53]. Furthermore, plankton density is reduced [e.g. 16, 45] impacting feeding mechanics of a filter feeder. *B. stygius* evolved to deal with these ecological challenges and the more complex oral sensory organs may be an example of an adaptation to these conditions.

### Variability among oral sensory organs

Given the histological resemblance of the different oral sensory organs, their variation in number of sensory cells was surprising. Not only did the numbers of sensory cells vary widely between 22 and 90, but there was also no symmetry between the left and right sides of the animal. While this could indicate individual variability, we do not know this for certain, because the specimen we analyzed histologically in detail is the first and only specimen of *B. stygius* available for such an analysis so far. The observation that the anterior left oral sensory organ contained three clusters of sensory cells could indicate a process of growth through multiplication, either during ontogeny or according to physiological needs. Alternatively, the three clusters of sensory cells could also represent an individual malformation. These explanations remain purely speculative until more specimens become available for microscopic analyses.

## Conclusions

Despite their importance as planktonic filter feeders, much needs to be learned about the diversity, disparity, and general biology of appendicularians in particular and marine invertebrates in general. A morphologically complex oral sensory organ with secondary receptor cells unique in tunicates, indeed in marine invertebrates, has been found in the present study in the mesopelagic giant appendicularian *Bathochordaeus stygius*. This surprising finding demonstrates once again the importance of exploratory and comparative research in order to understand biodiversity on an organismal level.

## Methods

Two specimens of *Bathochordaeus stygius* were provided by the MBARI (Monterey Bay Aquarium Research Institute; California USA) and collected via a remotely operated vehicle (ROV) *Ventana* in the Monterey Bay (California USA) using gentle suction [49]. Animals were fixed in a solution of 1% paraformaldehyde 2.5% glutaraldehyde in 0.2 M sodium cacodylate buffer (pH 7.2) adjusted to an osmolarity of approximately 800 mOsm with added NaCl. After one hour, primary fixation was stopped with three buffer rinses. Animals were kept in the same buffer and shipped via express mail to the Laboratory of Comparative Electron Microscopy at Humboldt University Berlin (Germany). Postfixation was then performed in a solution of 1% Osmiumtetroxide (OsO_4_) in double distilled water (ddH_2_O) and stopped with three rinses (once for 15 min and twice for 30 min) with ddH_2_O.

One specimen was embedded in Araldite after dehydration through a graded series of ethanol and subsequently used for histological analysis. Semithin sectioning of the entire trunk of this animal was accomplished via a Leica Ultracut S resulting in a transverse series of the animals’ trunk with 4529 sections of 2 µm thickness. Sections were stained with toluidine blue for 15–20 min. For reconstructions light microscopic images were recorded with a Canon EOS 700 D mounted on a Zeiss Axioplan compound microscope. To obtain an image stack of the entire trunk every 12th section was recorded (distance 24 μm) using the 2.5 × objective. For the reconstruction of the brain every section was recorded using the 40 × objective (distance 2 µm), every section for the exemplary reconstruction of one oral sensory organ, as well as every section for reconstruction of all nuclei of sensory cells in the remaining oral sensory organs using the 100 × oil-immersion objective (distance 2 µm). Reconstructions were manually completed in the software Amira 6.4.0 (Thermo Fisher Scientific) resulting in 3D anatomical models of the animal’s entire trunk, the brain and nerves as well as one oral sensory organ. 3D models of the brain and oral sensory organ were reconstructed on a cellular level, while trunk anatomy and organ systems were reconstructed on tissue level.

The second specimen was used for µCT-analysis in 70% EtOH in order to validate the morphological findings using an XRadia Versa 410 x-ray microscope (Carl Zeiss Microscopy GmbH Jena Germany). Micro-CT is a non-invasive imaging technique which allows for high-resolution and three-dimensional visualization of internal and external structures. Therefore, in addition to obtaining micro-CT data, we subsequently processed this second specimen for scanning electron microscopy. Briefly, the fixed specimen was cut in two halves along the mid-sagittal plane with the use of a scalpel. The two halves were dried through a graded series of ethanol and followed by critical-point drying in a Balzers Union CPD 030. The dried samples were mounted on a large SEM-stub sputter-coated with gold in a Balzers Union SCD 040 sputter coater and viewed with a LEO 1430.

### Supplementary Information

**Additional file 1. Figure S1**: Digital 3D reconstructions of the nuclei of the sensory cells in five of the oral sensory organs in one specimen of Bathochordaeus stygius as seen from dorsally. Notice the three clusters of nuclei in the anterior left oral sensory organ (osoal1-3). osoal/r—anterior oral sensory organ of the left/right side, osoll—lateral oral sensory organ of the left side, osoil/r—inner oral sensory organ of the left/right side.

**Additional file 2. Figure S2**: Light micrographs of cross sections through the approximate center of the epidermal opening of the anterior left oral sensory organ (osoal) in a specimen of Bathochordaeus stygius. aj—apical junctions, ci—cilia, ec—epidermis cell, ep—epidermis, ne—neurites, nu—nucleus, arrows—microvilli, asterisks—apical opening to exterior.

## Data Availability

All data are available from the corresponding author upon request.
